# Tumor Spheres Quantification with Smoothed Euclidean Distance Transform

**DOI:** 10.4172/2155-9937.1000143

**Published:** 2018-07-06

**Authors:** Ismet Sahin, Yu Zhang, Florencia McAllister

**Affiliations:** 1 Department of Engineering, Texas Southern University, Houston, TX, USA; 2 Department of Clinical Cancer Prevention, The University of Texas, MD Anderson Cancer Center, Houston, TX, USA

**Keywords:** Tumor sphere, Cancer stem cells, Segmentation

## Abstract

Tumor sphere quantification plays an important role in cancer research and drugs screening. Even though the number and size of tumor spheres can be found manually, this process is time-consuming, prone to making errors, and may not be viable when the number of images is very large. This manuscript presents a method for automated quantification of spheres with a novel segmentation technique. The segmentation method relies on initial watershed algorithm which detects the minima of the distance transform and finds a tumor sphere for each minimum. Due to the irregular edges of tumor spheres, the distance transform matrix has often more number of minima than the true number of spheres. This leads to the over segmentation problem. The proposed approach uses the smoothed form of the distance transform to effectively eliminate superfluous minima and then seeds the watershed algorithm with the remaining minima. The proposed method was validated over pancreatic tumor spheres images achieving high efficiency for tumor spheres quantification.

## Introduction

Cancer stem cells (CSCs) or tumor-initiating cells represent a subset of cancer cells with the potential to differentiate into the different sub clones existing in a tumor [1,2]. These cells also have metastatic capacity and are considered to represent an essential source for recurrent disease after cancer therapy [3,4]. CSC can form tridimensional spherical structures called tumor spheres (TS) when grown in non-adherent conditions in media supplemented with the required growth factors. The gold standard assay to characterize stem cell functionality is the TS formation assay which is based on the quantification of tumor spheres generated by plated single cells. However, this is a low yield assay since the rate of TS formation in this isolated condition is very low, therefore most TS formation assays are developed plating multiple cells [5,6] in multiple plates which is a time-consuming process. Therefore, automatization of spheres counting after images acquisition has been encouraged especially for drug screening assays in which thousands of wells are analyzed simultaneously. Image segmentation has been applied to problems in pattern recognition, computer vision, and medical imaging [7,8]. Segmentation is the process of splitting gray or colored digital image into different regions where each region contains elements with similar characteristics. The location and size of these regions carry important information for many applications and it is fundamental for optimal image automated quantification. In the current manuscript, our main interest is to improve image segmentation for automated tumor spheres quantification.

The watershed construction is a well-known image segmentation algorithm [9,10]. This algorithm views a two-dimensional image as a three-dimensional image where the third dimension is the gray intensity level. If a pixel has the lowest intensity level within its neighborhood, then it is called a local minimum. There are often many local minima in a given image. A watershed algorithm starts filling water into the basins of these minima and whenever water from two basins is about to mix, the algorithm constructs a wall (shed) between these two basins, effectively preventing water from mixing. This process continues until the highest value in the image is reached and results into the walls between the basins. These walls become the borders of the segmented spheres. However, there are usually more than one minimum within a single tumor sphere and therefore, the watershed algorithm splits the sphere into many superfluous regions [10]. This manuscript proposes an algorithm that aims to resolve this over-segmentation problem. The major step of the algorithm is low-pass filtering of the distance-transformed image. Filtering reduces the number of minima within each tumor to one minimum and using the watershed algorithm receives this filtered output and produces the correct sphere borders.

## Materials and Methods

This section describes the proposed image segmentation method for automated identification of tumor spheres. In the first step, a color image is converted into image.

$$I_{1}=0.2989\cdot R+0.5870\cdot G+0.1140\cdot B$$

I_1_ with gray intensities by the following weighted averaging of the red (R), green (G), and blue (B) channels: Where the coefficients are default values used in the MATLAB image processing toolbox. When border pixels have similar gray levels to the tumor spheres, incorrect identification of spheres may occur. Two lines of pixels from each edge of the image I_1_ are removing to avoid this problem.

A two-dimensional Gaussian filter is applied to I_1_ for obtaining its smoothed form. This operation reduces the sharp variations within the background and tumor sphere regions and provides a clear separation of these two regions. The Gaussian distribution is given by

(2) $$G(x,y)=1∕(2\pi \sigma^{2})e^{\frac{x^{2}+y^{2}}{2\sigma^{2}}}$$

where σ denotes the standard deviation of the distribution. A Gaussian kernel *hG* is formed by sampling *G* (*x, y*) with a specific standard deviation σ1. Convolution of the original image *I*_1_ with this kernel yields the smoothed image *I*_2_:

(3) $$I_{2}=I_{1}*\mathrm{hG}(\sigma_{1})$$

Heterogeneous illumination during the image acquisition causes considerable amount of variation in the image background where the corners are darker than central parts. An image with homogeneous background is obtained by using a similar approach to the one described by Bowman’s et al. This approach involves a square window *W_ij_* centered at the pixel (*i*, *j*):

(4) $$W_{\mathrm{ij}}=\{(x,y):\;|x-i|\;\leq l∕2,\;|y-j|\;\leq l∕2\}$$

where *i* = 1,…, *M* and *j* = 1,…, *N* as the image size is *MxN*. Notice that each side of this square is *l* pixels. The mean intensity *μ_ij_* of the pixels within the window is found:

(5) $$\mu_{\mathrm{ij}}=\frac{1}{I_{2}}\sum \begin{array}{c} (x,y)\varepsilon w_{\mathrm{ij}} \end{array}I_{2}(x,y)$$

The mean intensity represents the estimated background at the specified pixel, that is *B* (*i*, *j*) = *μ_ij_*. The image *I*_3_ with the homogeneous background is determined by subtracting the background image B from the smoothed image *I*_2_:

(6) $$I_{3}=I_{2}-B$$

The window centered at an edge pixel includes an area that is outside of the image. The gray level of each pixel within this area is considered to have the same gray level with the nearest pixel on the edge. This method is effective replicating boundary pixels whenever extrapolation is needed. The Otsu’s method is then applied to the image *I*_3_ for converting the gray image into a binary image. This method finds the optimal threshold *rotsu*:

(7) $$\mathrm{rotsu}=\mathrm{Fotsu}(I_{3})$$

where *Fotsu* (·) is the Otsu’s method [Ots1979, Bou2014]. The threshold splits the background from the foreground in a way that the weighted sum of intra-group variances is the smallest. This is equivalent to having the largest inter-variance between the foreground and background. The threshold function *T* (·) yields the binary image I_4_:

(8) $$I_{4}=T(I_{3},\mathrm{rotsu})$$

The image *I*_4_ has white pixels representing the tumor spheres and black pixels representing the background. Small patches of black pixels within the tumor spheres are converted to white pixels by using the morphological operation of filling.

In the next step we perform the Euclidean distance transform to *I*_4_ that assigns the smallest distance from each pixel of the tumor spheres in *I*_4_ to the nearest background pixel. More specifically distance *dij* from a pixel *p_ij_* of the tumor sphere to the nearest background pixel *b* is given as follows

**Figure 1:**
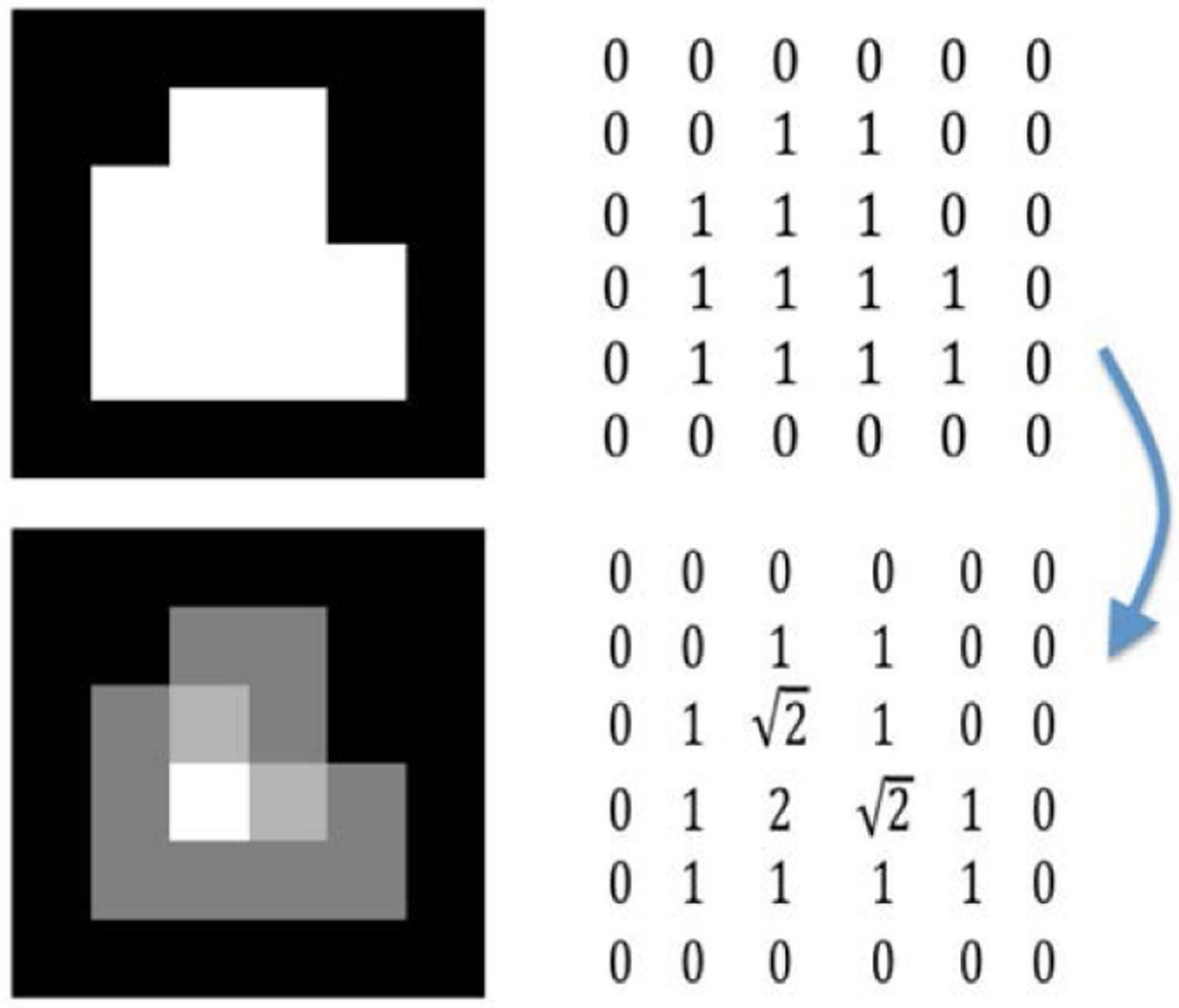
First row shows the original image and the corresponding matrix while the second row shows the transformed image and the corresponding matrix.

**Figure 2:**
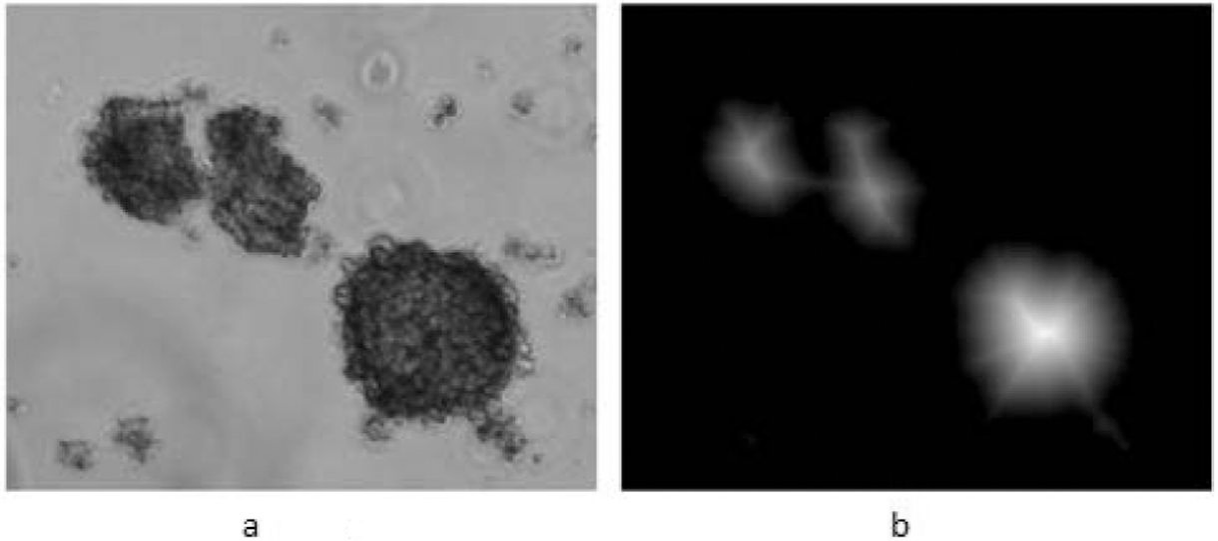
**(a)** The original image and **(b)** the Euclidean distance-transformed form of the image.

(9) dij=db∈Bmin(pij,b)

where *d* (*x, y*) is the Euclidean distance between two points *x* and *y*. The distance transformed image *I*5 is constructed by assigning this distance as the element on the *ith* row and *jth* column: *I*_5_(*i*, *j*) = *d_ij_*. An example with a simple 6 × 6 binary image is illustrated in Figure 1. The original binary image and its transformed image are under the first column while their matrix representations are presented under the second column. The brightest pixel of the transformed image is the farthest pixel from the edges of the white region, representing the center of the tumor sphere in this case. A real case is demonstrated in Figure 2 where:

(a) shows an original image with three tumor spheres while,
(b) shows the distance-transformed image.

If the shape of a tumor sphere deviates from a circular shape largely or its border makes large wiggles, then there may exist two or more local maxima within the sphere border since the level of brightness depends on the nearest distance to the background. An example of this can be observed in Figure 2b where the middle tumor sphere is elongated almost vertically, and one maximum is located on the top part and the other maximum is located on the bottom part of the sphere. Since this sphere has two local maxima and since each maximum is associated with a tumor sphere, this sphere would be segmented into two separate superfluous spheres by the watershed algorithm.

To overcome this problem, we smoothen the distance-transformed image to eliminate high frequencies in spatial domain. The higher spatial frequencies are discarded from the distance-transformed image I5 after passing I_5_ through a low-pass filter. Since the Fourier transform of a Gaussian waveform is another Gaussian that decays smoothly without ripples in the frequency domain, convolution in spatial domain effectively eliminates the higher spatial frequencies. This is the low-pass filtering operation: We filter I_5_ with a Gaussian hG(σ2) in the spatial domain:

(10) $$I_{6}=I_{5}*\mathrm{hG}(\sigma_{2})$$

where * represents the convolution operation and hG(σ2) is a Gaussian kernel. The sigma parameter, σ2, of the Gaussian needs to be determined for eliminating high spatial frequencies. When σ2 is too small, the filter hG(σ2) only stops very high spatial frequencies and as a result I_5_ is not smoothed enough and some superfluous spheres may remain. In other words, over-segmenting problem persists with a small σ2. On the other hand, when σ2 is too large, I_5_ is smoothed largely and as a result true spheres may be fused into a single sphere. In this case we have the problem of under segmenting. By experimental iteration we found that low pass filtering with the sigma values between 5 to 11 provides optimal segmentation results and therefore we chose σ2 = 8 which is in the center of this range of values. The resulting image *I*_6_ is the smoothed distance-transform matrix without superfluous maxima that existed in *I*_5_. Watershed construction algorithm finds the sphere boundaries based on the image *I*_6_.

**Figure 3:**
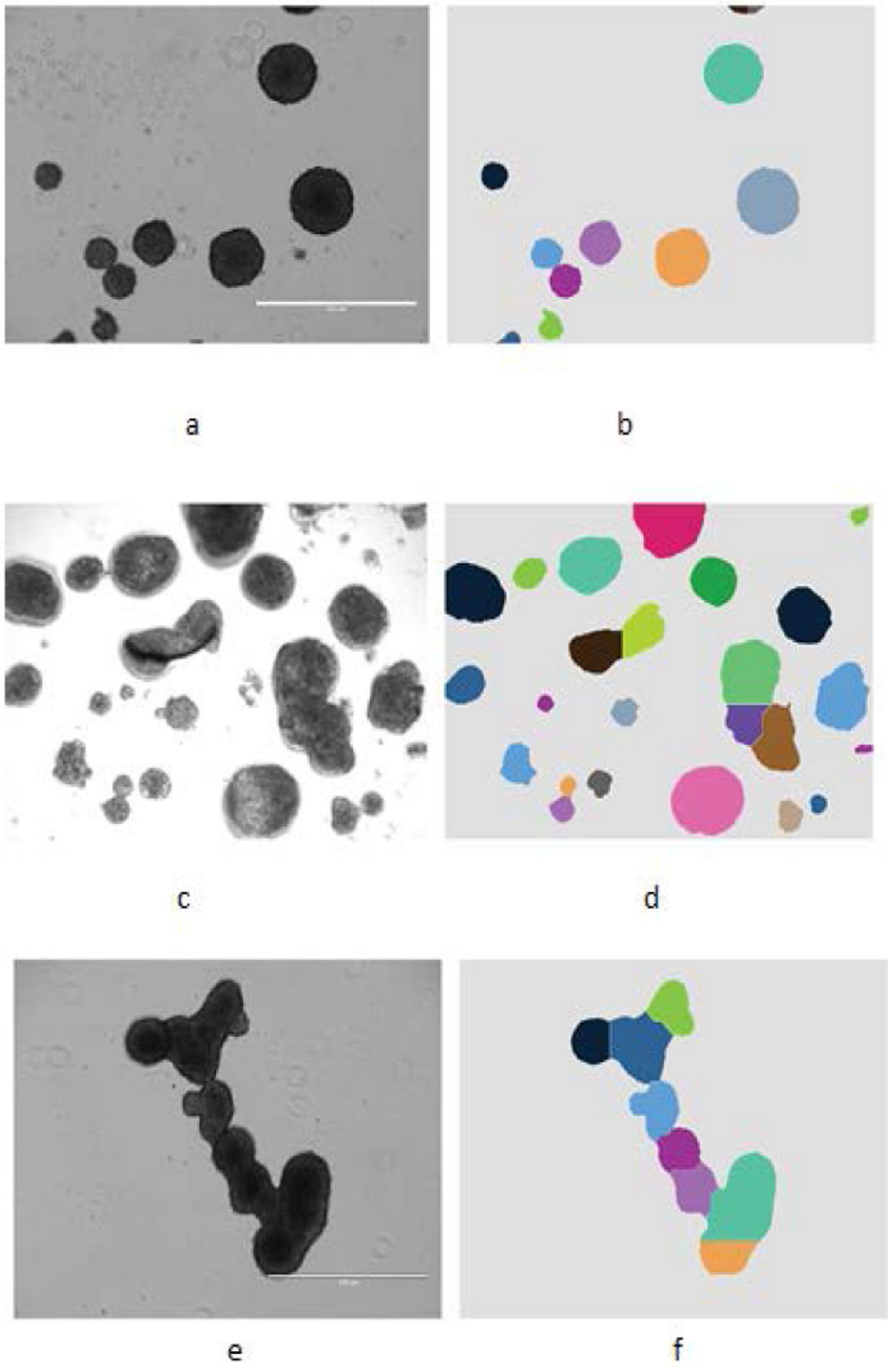
**(a, c, e)** Original images and **(b, d, f)** Segmented images respectively.

This image is first negated to make maximum points to be minimum points. In other words, the centers of the spheres are the highest points which are turned into the lowest initial points for the watershed algorithm. Starting with these sphere centers, the watershed algorithm yields accurate results as is demonstrated in Figure 3. Three original images are under the left column and the corresponding segmented images are under the right column. Segmentation of the image in Figure 3a was relatively easy since:

(i) Almost no spheres are overlapping,
(ii) Background is relatively homogeneous, and
(iii) Spheres are shaped circularly.

Figure 3b demonstrates that segmentation of two or three overlapping spheres was successful. The spheres with relatively non-circular shapes are also identified correctly. Figure 3b shows the segmentation result for a more challenging image where spheres are largely overlapped. To evaluate the performance of the proposed algorithm, a tumor sphere assay was set up in 96 separate wells as usually done for drugs screening. Ten microscopy-based pictures of the initial 10 wells are presented in this report and the number of tumor spheres is counted manually and automatically with the proposed method. Spheres size was also recorded during the automated counting. The manual count specifies the average of two expert’s manual counts. The counting results are listed in Supplementary Table 1. The proposed method achieves a high level of accuracy as the automated counts and manual counts are either the same or they differ by 1 for first 9 images. The overall automated and manual counts are 102 and 99 where the difference is about 3%. Experimental duplication has achieved similar results. The algorithm also lists the size of each tumor sphere in any given image. As an example, we presented the sphere sizes for the image in Figure 3d in Supplementary Table 2.

## Result and Discussion

Automated quantification of tumor spheres is of great interest to biologist as it can save their time and effort while achieving high quantification accuracies. Extended period of manual counting sessions is also prone to making more human errors, which can be eliminated with automated quantification which primarily involves image segmentation. Multiple methods have been developed to reduce the over segmentation problem with the watershed algorithm used for automatic segmentation for images like the ones containing tumor spheres. Parvati et al. have used morphological operations of erosion and dilation to mark the foreground regions before applying watershed algorithm [11]. Cheng and Rajapakse use shape markers in the watershed algorithm for segmenting nuclei of cells in fluorescence microscopy images [12].

## Conclusion

In the present manuscript, instead of depending on the features obtained from the original distance transform matrix, we apply smoothing filters to the distance transform which effectively replaces superfluous minima with locally averaged distance values. This technique makes these minima disappear in the smoothed form of the distance transform. We demonstrated using medical images that applying the watershed algorithm to the smoothed distance transform matrix achieves high efficiency for tumor spheres quantification.

## Supplementary Material

Suppl File
