# Supplementary material for: Tumor Spheres Quantification with Smoothed Euclidean Distance Transform
Source: J Mol Imaging Dyn. Author manuscript; Available in PMC 2018 Oct 10. (PMC6179360; doi:10.4172/2155-9937.1000143)
Supplement: Suppl File [file NIHMS987248-supplement-Suppl_File.pdf]

Image Number	Automated Count	Manual Count	ACT	MCT
1	17	17	0.28	6.27
2	19	20	0.25	7.11
3	23	22	0.29	7.32
4	6	6	0.25	2.01
5	3	3	0.66	0.43
6	8	8	0.68	1.54
7	11	10	0.68	2.52
8	3	3	0.62	0.54
9	5	5	0.63	1.45
10	7	5	0.62	1.39
Total	102	99	4.96	30.58

Table 1: Comparison between automated counting time (ACT) versus manual counting time (MCT).

Sphere Number	Area
1	2168
2	10586
3	9556
4	7339
5	1907
6	4433
7	2174
8	25677
9	16853
10	4433
11	5348
12	14835
13	28278
14	36762
15	16041
16	29854
17	11406
18	20683
19	5527
20	21671
21	2308
22	25249
23	2457

Table 2: The areas of the spheres in Figure 3d quantified with the proposed algorithm (area is measured in um^2).
